# A Cone-Beam Computed Tomography Study of the Morphological and Morphometric Variations in the Mandibular Lingula and Its Clinical Implications

**DOI:** 10.3390/diagnostics15162071

**Published:** 2025-08-18

**Authors:** Hui Wen Tay, Wei Cheong Ngeow

**Affiliations:** Department of Oral & Maxillofacial Clinical Sciences, Faculty of Dentistry, Universiti Malaya, Kuala Lumpur 50603, Malaysia

**Keywords:** mandibular lingula (ML), cone-beam computed tomography (CBCT), sagittal split ramus osteotomy (SSRO)

## Abstract

**Background/Objectives:** The mandibular lingula (ML) is a small bony projection on the medial surface of the ramus and serves as the first reference point identified during sagittal split ramus osteotomy (SSRO) or inferior alveolar nerve block (IANB). Anatomical variations in the mandibular ramus have been shown to exist across different populations. Understanding these population-specific differences enhances both clinical safety and diagnostic precision. However, there is a paucity of anthropological data amongst the Mongoloid population, especially in Southeast Asia. Hence, this study aimed to investigate the (i) distance of the lingula to different mandibular anatomical landmarks and its localization, (ii) lingula shape, and (iii) differences between gender and the sides of the mandible amongst the local ethnic groups. **Methods**: This retrospective cross-sectional study consisted of 206 cone-beam computed tomography (CBCT) images of 77 males and 129 females (mean age 33), with a total of 412 hemimandibles. Measurements were performed on three-dimensionally reconstructed CBCT images. **Results**: The most common shape was the truncated type. The distance of the lingula to the anterior (LiA), posterior (LiP), superior (LiS), and inferior (LiI) borders of mandible were 17.84 (2.25) mm, 14.46 (3.44) mm, 17.73 (3.00) mm, and 27.05 (4.40) mm, respectively. No significant difference exists between the sides of the mandible. Sexual dimorphism existed for all lingula measurements except LiA. Indians have smaller rami with more anteriorly and inferiorly placed ML than Malay and Chinese. The majority of ML was located 8.55 mm above the occlusal plane. **Conclusions**: This study provides information on the ML and its surrounding ramus structure in the local population. Such variations must be accounted for in SSRO and IANB.

## 1. Introduction

The mandibular lingula (ML) is a small, sharp bony projection on the medial surface of the mandibular ramus that is closely related to the mandibular foramen (MF) through which the inferior alveolar nerve enters and courses through the canal. Lingula, due to its tongue-shaped morphology, was aptly derived from the Latin word “Lingua”, which meant “little tongue”.

Clinically, the mandibular lingula is an important anatomical landmark in procedures where the mandibular foramen needs to be identified, such as the inferior alveolar nerve (IAN) block [1] in mandibular anesthesia and sagittal split ramus osteotomy (SSRO) in orthognathic surgery [2]. In medial horizontal osteotomy for SSRO, ML is the first anatomical reference point to be identified [3], while mandibular anatomical variation has been attributed as one of the factors [4] for IAN block failure, which was reported to be as high as 10 to 15% [5].

Several authors have investigated the mandibular foramen (MF) [6,7,8]. However, intraoperatively, it may prove difficult for surgeons to identify the mandibular foramen directly due to limited surgical access, low surgical field of vision, natural anatomical concavity of the mandibular ramus, and the presence of musculotendinous attachment, thus limiting its clinical applicability. On the other hand, as the ML appears as a bony protuberance on the ramus medial surface, it allows identification and localization more readily by surgeons, either by direct visualization or through tactile sensation. Studies have established the reliable and stable relationship of ML with the MF [9]. According to Tengku Shaeran et al. [10], the MF is situated inferiorly and posteriorly to the greatest prominence of the ML.

To date, the literature has reported on four cardinal shapes of the ML, including triangular, nodular, truncated, and assimilated [11], while a few other studies reported on a fifth shape described as the “M” shape [12,13], “bridge” shape [14], and mixed type [15]. The exact reason for the variability in the shape of the lingula remains unknown. Table 1 summarizes the current literature on the morphology of ML.

In addition to morphological variations, variable morphometric data of the ML have also been reported in the Turkish [24,28,35,37], Thai [16], Taiwanese [38], Indian [19,25,26], Korean [39], Saudi Arabian [9,36], Brazilian [3,27,29], Italian [40], and Kenyan [41] populations. These studies used either dry mandibles, computer tomography (CT), or cone-beam computed tomography (CBCT) imaging for the anatomical assessment and description of the ML.

CBCT, a useful diagnostic imaging modality, was used in this study due to its high geometric accuracy and spatial resolution; in-office accessibility; and lower radiation dose compared to computed tomography (CT).

Although the ML and its surrounding anatomical ramus structures have been extensively studied, local data in the Mongoloid population, particularly the Malaysian ethnic groups, remain scarce in the present time. The failure of surgeons to identify and recognize these morphological and morphometric variations may lead to an increased risk of complications such as neurosensory disturbances, intraoperative bleeding, and poor split. Hence, this study aimed to investigate the morphological and quantitative parameters of the ML, including the (a) shape; (b) height; (c) distance to the occlusal plane; (d) distance to the ramus borders (anterior, posterior, superior, inferior); and its association. The presence of any significant differences between the sides of the mandible, gender, and ethnicities were also investigated.

The clinical relevance and significance of the data obtained from this study include providing a safe guide for surgeons when performing the medial horizontal osteotomy in SSRO, making it more predictable and reliable with fewer complications of IAN injury and poor split. The data amongst the local ethnic groups may also provide added anthropological value and contribute to the existing literature.

## 2. Materials and Methods

This retrospective cross-sectional study was conducted at the Department of Oral and Maxillofacial Clinical Sciences, Faculty of Dentistry, Universiti Malaya, from July 2022 to August 2023, with ethical approval received from the Medical Ethics Committee, Faculty of Dentistry (protocol reference number: DF OS2207/0013 P). A total of 471 CBCT images of patients who presented to the Oral and Maxillofacial Surgery Clinic and had CBCT imaging taken for different reasons including impacted teeth, implant surgeries, orthognathic surgeries, and maxillofacial fractures at the Oral Radiology Unit, Faculty of Dentistry, Universiti Malaya, between 2011 and 2015 were screened. This collection of CBCTs has been used in various anatomical studies previously; but no newer CBCTS were taken after the machine malfunctioned in 2015, thus limiting our availability of newer data. All patients whose CBCT data and records were used in this study provided prior written consent agreeing to release their data for research or academic purposes. No CBCT imaging was taken solely for research purposes.

Inclusion and exclusion criteria

The inclusion criteria were as follows:

1. Malaysian subjects whose ethnicities were either Malay, Chinese or Indian;
2. Age between 18 and 60 years old;
3. Presence of first molars on both sides of the mandible.

The exclusion criteria consisted of the following:

1. Subjects with poor quality CBCT with distortion of anatomical reference landmarks;
2. Edentulous mandible;
3. Presence of dentofacial deformities involving the mandible (i.e., syndromic patients);
4. Presence of pathologic lesions such as cyst or tumour, mandibular fractures;
5. Patients with a history or evidence of surgical intervention to the mandible.

The independent variables, including the demographic background such as age, gender, and ethnicity were collected from the patient registration records system. The primary outcomes were the different mandibular ramus anatomical measurements. The secondary outcomes were the differences between gender, the sides of the mandible, and ethnicities.

Sample size calculation was based on the prevalence of 15.8% (*p* = 0.16) from a previous study by Tuli et al. (2000) [11] and a level of confidence set at 95% (z = 1.96 for standard normal distribution) and a margin of error at 5% (e = 0.05). This yielded 205 samples needed. Consecutive patients who fulfilled the inclusion and exclusion criteria were selected and a final sample of 206 CBCT images were included in this study. The right and left sides of each jaw were evaluated individually, corresponding to a total sample of 412 hemimandibles.

All the CBCT scans were acquired using i-CAT Vision System by Imaging Sciences International Inc. (Hatfield, PA, USA), operating at 120 kVp, 5–8 mA with an exposure time of 20 s. Only CBCTs with a field of view of 16 cm in diameter and 13 cm in height with a 0.25 mm voxel size were included. All images were taken by the same radiographer according to a standard protocol and patient positioning. The original slice image CBCT data acquired from the proprietary manufacturer acquisition software, i-Cat Vision (Imaging Sciences International Inc., Hatfield, PA, USA), was extracted and saved in Digital Imaging and Communications in Medicine (DICOM) multi-file format and exported to 3D Slicer software (3D slicer project by Brigham and Women’s Hospital, BWH, Inc. supported by NA-MIC, NAC, BIRN, NCIGT, and the Slicer Community, Boston, MA, USA). The DICOM data were then converted and reconstructed into 3-dimensional (3D) surface rendered volumetric images of the mandible using the 3D Slicer software Version 5.0.2 (https://www.slicer.org).

To reduce bias and errors, all CBCTs were reviewed and evaluated on one independent workstation by the same researcher using standardized pre-adjusted image contrast and brightness on a MacBook Air 13.3-inch with Apple M1 chip (8-Core CPU and 7-Core GPU and 2560 × 1600 native resolution at 227 pixels per inch) manufactured by Apple Inc., Cupertino, CA, USA. Anatomical landmark identification and localization, the setting of reference planes, and direct linear measurements on the 3D volumetric reconstructions were performed by the same principal investigator with 9 years of clinical experience. The investigator was trained and calibrated with a consultant in the oral and maxillofacial surgery with more than 20 years of experience in similar research prior to the commencement of this study.

Shape of lingula

**Figure 1:**
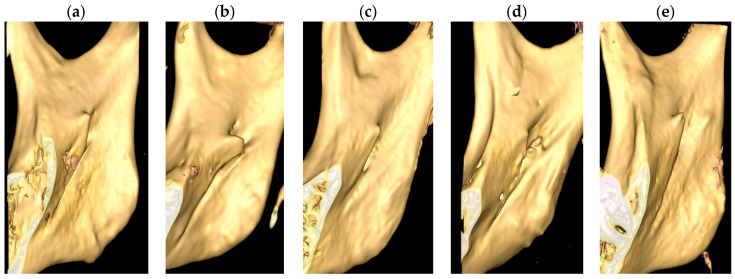
Five shapes of lingula. (**a**) Triangular. (**b**) Truncated. (**c**) Nodular. (**d**) Assimilated. (**e**) Others.

The shapes of ML were classified according to the classification by Tuli et al. (2000) [11], with the addition of a fifth group categorized as “others”. Morphology of the lingula, which did not fit into either one of the four conventional types, was classified under “others” (Figure 1):

1. Triangular: Broad-based and pointed apex;
2. Nodular: Rounded apex;
3. Truncated: Flat projection with blunt upper margin;
4. Assimilated: Completely incorporated into ramus of mandible;
5. Others: M-shaped.

Reference plane

Prior to measurements, 3D spatial positions of all constructed mandibular models were standardized, with two reference planes set up. The occlusal plane (OP) was made the horizontal reference plane, while the plane perpendicular to the horizontal occlusal plane was established as the vertical reference plane.

**Figure 2:**
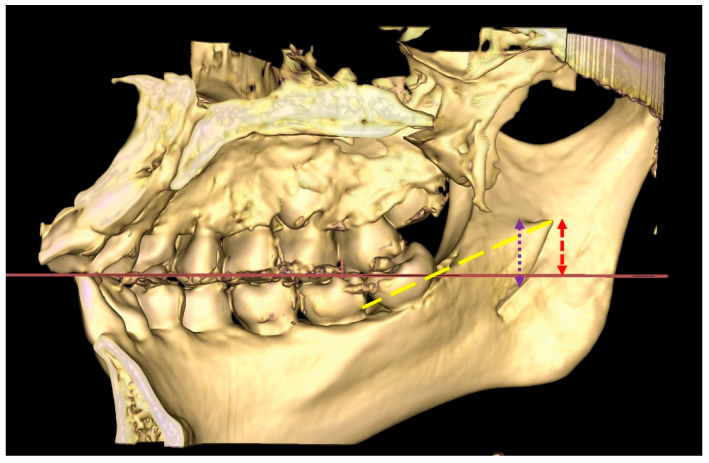
Occlusal plane (OP) as the horizontal reference plane (red horizontal line); distance of lingula tip to M2M, LIM2M (yellow dashed line); HLi (purple dotted arrow); LiOP (red dashed arrow).

After the horizontal and vertical reference planes were established, the anatomical landmarks were identified and marked, as described in Table 2 and illustrated in Figure 2.

**Figure 3:**
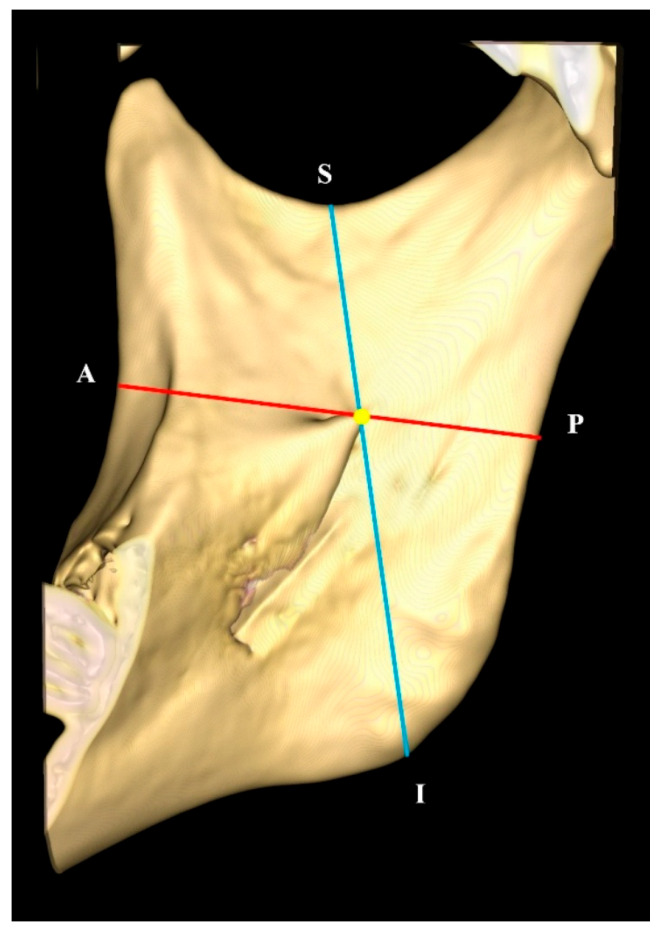
Anatomical landmarks and lingula measurements on the 3D mandibular models. Point A: deepest concavity at the anterior border of ramus. Point S: deepest concavity of the sigmoid notch at the superior border of the ramus. Point P: posterior border of ramus. Point I: inferior border of ramus.

Linear measurements from the tip of the lingula to the superior, inferior, anterior, and posterior borders of the ramus of the mandible were performed on the medial surface of the 3D reconstructed mandible (Figure 3). To localize and determine the antero-posterior and supero-inferior position of the lingula, the ratios of ‘LiA’ to ‘LiP’ and ‘LiS’ to ‘LiI’ were calculated, respectively. A smaller ratio would indicate that the mandibular lingula was located at a more anterior and superior portion of the ramus of the mandible.

For the assimilated type of lingula, which was incorporated entirely into the ramus with no visible tip, measurement was performed from the upper- and anterior-most of the mandibular foramen to the different mandibular anatomical landmarks.

Detailed descriptions are summarized in Table 3.

Reliability of measurement

Thirty CBCT scans were randomly selected to repeat all the measurements after a period of 30 days to assess for intra-observer reliability. The intraclass correlation coefficient (ICC) showed Cronbach’s alpha value of 0.803, suggesting good reliability.

Statistical analysis

All data were analyzed using SPSS version 20.0 (IBM Corp., Armonk, NY, USA). For the shape of the lingula, the data were presented in frequencies (*n*) and percentages (%). For each measurement, the mean, standard deviation (SD), and maximum and minimum values with a 95% confidence interval (CI) were calculated and tabulated. Normality of data was assessed using the Kolmogorox–Smirnov test. An independent t-test was used to compare the difference between gender (male and female) and the sides of the mandible (right and left sides). A one-way ANOVA test was used to compare the differences between ethnicities (Malay, Chinese, Indian). A *p*-value < 0.05 was considered statistically significant.

## 3. Results

**Figure 4:**
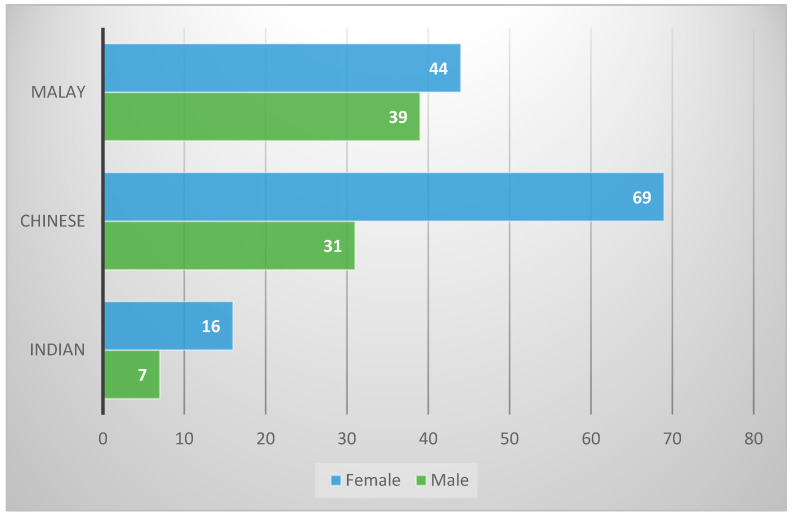
Distribution (frequency, *n*) by gender and ethnicity.

CBCTs of 206 patients with a total of 412 hemimandibles were evaluated. The mean age of the study population was 33.26 ± 9.93 years. There were more females (*n* = 129, 62.6%) than males (*n* = 77, 37.4%) and the detailed demographic distribution is shown in Figure 4.

### 3.1. Shape of Lingula

**Figure 5:**
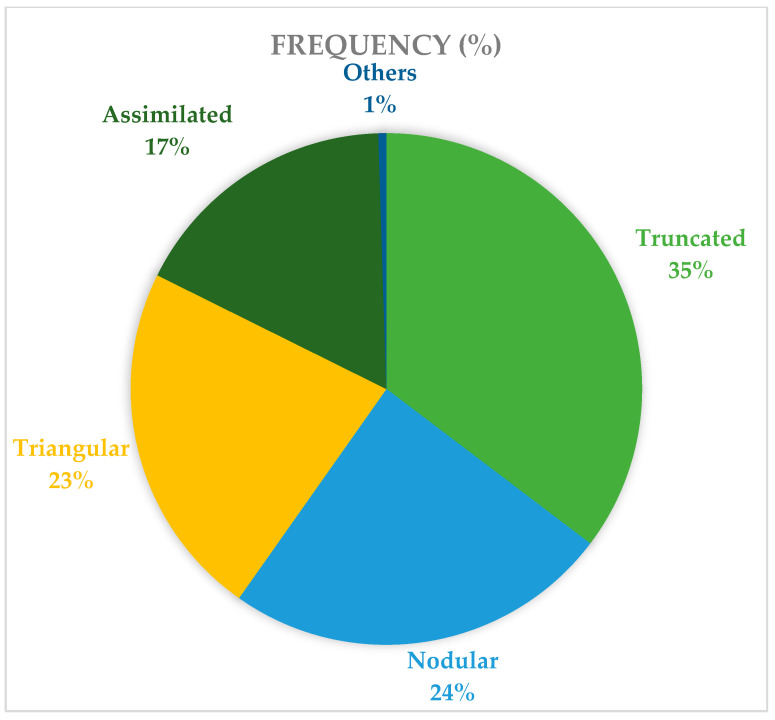
Frequency (in percentage, %) of lingula shape.

There were no significant differences between the right and left sides (chi-square test; *p* = 0.962) and between genders. Truncate-shaped ML was the most common appearance, accounting for 146 (35.4%) of the total 412 lingulae studied, followed by the nodular (*n* = 101; 24.5%) and triangular (*n* = 93, 22.6%) types, with the least being the assimilated type (*n* = 71; 17.2%) (Figure 5). There was a fifth morphological variant observed at the right side of the mandible of a Malay male (0.2%). Overall, the truncate-shaped lingula was the most common type of lingula in all ethnic groups (Malay—*n* = 57; 34.3%; Chinese—*n* = 68; 34.0%; Indian—*n* = 21; 45.6%). The chi-square test showed a significant difference (*p* < 0.05) between ethnicities for the shapes of the lingula on both the right (*p* = 0.006) and left side (*p* = 0.02) of the mandible. Bilateral distribution (presence of the same morphological variant on both sides of the mandible) is slightly more than unilateral distribution (Table 4).

### 3.2. Height of Lingula (HLI)

No significant differences between the right and left sides and between genders exist with regards to the HLi (Table 5). Out of the 412 hemimandibles in this study, 71 assimilated types (absence of lingula), with no recorded height, were excluded, giving a total of 341 lingulae with a mean height of 5.25 (1.36) mm. The height of the lingula of the Chinese [5.29 (1.38) mm] > Malay [5.26 (1.33) mm] > Indian [4.95 (1.33) mm], with no significant difference between ethnicities.

### 3.3. Distance of Lingula Tip to the Occlusal Plane (LiOP)

Similarly, LiOP showed no significant differences between the right and left sides and between genders. Out of the 412 hemimandibles in this study, 393 lingulae were found to be located above the occlusal plane (OP), 15 lingulae at the same level as the occlusal plane (distance = 0 mm), and 4 were below the occlusal plane. The total mean distance of 393 lingulae above the occlusal plane was 8.55 (3.34) mm (Table 6). The four lingulae below the occlusal plane reported a mean of 2.93 (0.70) mm. One-way ANOVA showed significant differences (*p* = 0.011) between the Malay and Indian (*p* = 0.035) and Chinese and Indian (*p* = 0.033) for LiOP distance.

### 3.4. Distance of Lingula Tip to the Mandibular Second Molar (LiM2M)

Forty-six hemimandibles had a missing mandibular second molar (M2M); hence, the mean LiM2M obtained from 366 hemimandibles was 31.47 (3.53) mm (Table 7). Males generally have a larger distance of lingula to M2M than females in all ethnicities. Significant differences exist only between Malay and Indian (*p* = 0.003) and Chinese and Indian (*p* = 0.016).

### 3.5. Distance of Lingula Tip to Anterior (LiA), Posterior (LiP), Superior (LiS), and Inferior (LiI) Borders of Ramus

The overall distances of lingula to the anterior (LiA), posterior (LiP), superior (LiS), and inferior (LiI) borders of the ramus were 17.84 (2.25) mm, 14.46 (3.44) mm, 17.73 (3.00) mm, and 27.05 (4.40) mm, respectively. In general, males have larger ramus than females (Table 8). Only the distance of the lingula to the anterior border of the ramus (LiA) showed no gender dimorphism (*p* = 0.713). Significant differences existed between ethnicities for LiA, LiS, and LiI (*p* < 0.05), except LiP (Table 8).

The mean LiA: LiP and LiS: LiI ratios were 0.56 (0.06) and 0.40 (0.05), respectively, with statistical significance between gender but not ethnicity. The LiA: LiP for males (0.54) was higher than females (0.56), suggesting that males have more anteriorly placed lingula in the mid-quadrant.

**Figure 6:**
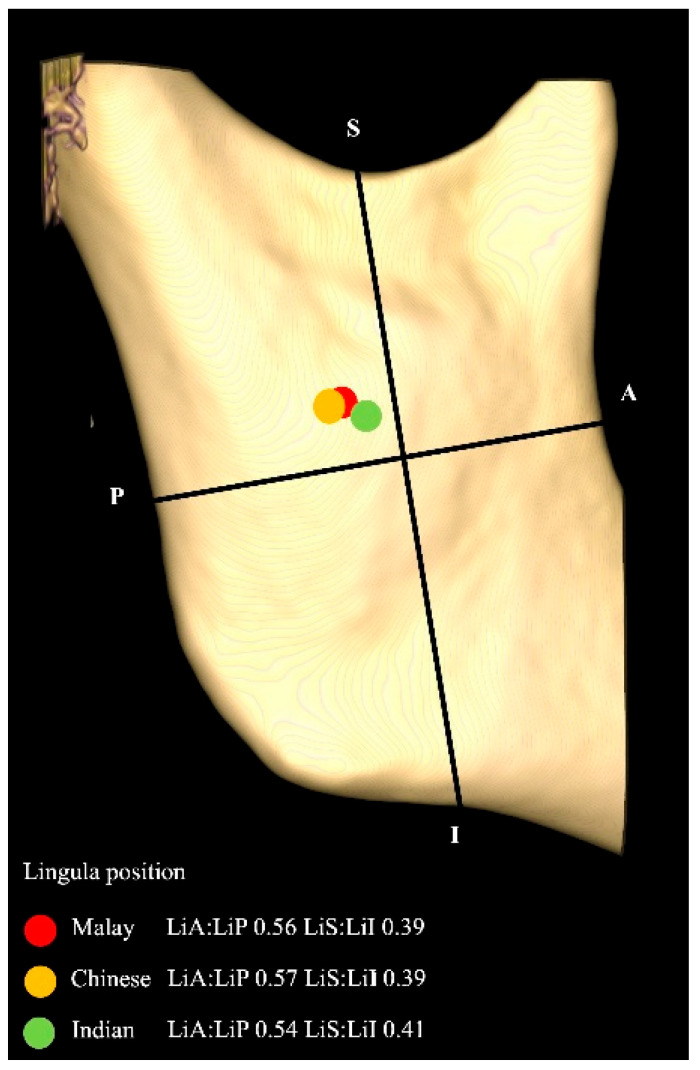
Localization of ML in relation to the mid-ramus according to ethnicity.

Overall, it can be noted that the Chinese showed the highest measurement for LiA and the lowest measurement for LiS, suggesting that their lingula was higher and the most posteriorly placed among the three ethnicities. The Indians, on the other hand, showed the largest distance for LiS and the shortest distances for LiA, LiP, and LiI, suggestive of a smaller ramus, with a lower lingula. Figure 6 showed the localization of lingula in relation to the mid-ramus for the different ethnicities.

## 4. Discussion

Knowledge about the morphometric relationships between the ML and the surrounding landmarks may allow localization of this structure more effectively especially when it is absent or in centres where advanced 3D imaging is a luxury.

Several studies have been performed in the Asian region on Thai [16], Taiwanese [38], Indian [19,25,26], Korean [39,42], and Malay [41] populations but this is the first local study carried out to compare the mandibular lingula (ML) amongst the three main ethnicities representing the Malay, Chinese, and Indians in Malaysia.

Shape of Lingula

The mandibular lingulae were not sufficiently and precisely described until the year 2000, when Tuli et al. classified the varying morphological shapes into triangular, truncated, nodular, and assimilated type. Since then, this classification has been widely used by subsequent mandibular lingula studies among different populations. Similarly, in the present study, the classification system by Tuli et al. [11] was adopted, but with an additional fifth shape observed, the ‘M’ shape, also described by Assis et al. (2019) [12] and Varma et al. (2013) [13] in the Indian population.

The ML has been extensively studied, particularly in India, where most studies involved the direct visualization of lingula shape on dry mandibles. Amongst the Indian population, the triangular morphology was the most common shape except for studies conducted by Varma et al. (2013) [13] and Padmavathi et al. (2014) [25], where the nodular and truncated types were the most prevalent, respectively. Our current findings on Malaysian Indians differ from those reported in most previous studies, but given the small sample size of 23 subjects, this may not represent the whole population.

A recent systematic review and meta-analysis of 4694 subjects revealed the triangular shape to be the most common [43]. In contrast, the truncated type was the most prevalent in the Italian [14], Thai [16,30], and Brazilian [27] populations, which was similar to the finding in this study amongst the Malaysian population. Another finding, consistent with previous studies, is the prevalence of the assimilated type (absence of the lingula), which is as low as 1–4% [14,24,44]. However, ethnicity-wise, the prevalence of the assimilated type is relatively high among the Malaysian Malay (21.7%) and Indian (34.8%).

There is a lack of evidence on the clinical significance or impact of lingula shape on procedures such as sagittal split ramus osteotomy (SSRO) or inferior alveolar nerve block (IANB). Nonetheless, it is postulated that the triangular shape, which is the most prominent, would be the most readily identifiable and detectable during SSRO. In addition, a recent paper also suggested that the shape of the lingula may influence its position within the ramus [44].

HLi, LiOP, LiM2M

Based on a meta-analysis of 4694 subjects, the mean height of ML was 8.17 ± 0.22 mm; larger in cadavers (8.26 ± 0.39 mm) than that in imaging studies (7.84 ± 0.10 mm) [43].

The overall height of the lingula (5.25 mm) in this study was comparatively lower, with no significant differences between sides, gender, or ethnicities. The HLi between the males and females was comparable at 5.23 mm and 5.26 mm, respectively. This contrasts with the findings of a few studies [9,24,27,38], which reported greater lingula height in males than in females. The mean height of the lingula on the left side (5.33 mm) was slightly higher than the right side (5.17 mm) of the mandible. This may be due to the assimilated type of lingula, with no recorded height, being found more frequently on the right side compared to the left side in this study. As for ethnicity, the HLi in descending order was Chinese (5.29 mm) > Malay (5.26 mm) > Indian (4.95 mm), which could be attributed to the higher number of assimilated lingula found in Indians.

The clinical significance of this parameter lies in the presence of a safety margin, especially in lingulae with greater height. In sagittal split ramus osteotomy based on Hunsuck’s modification [45], the placement of medial horizontal osteotomy is determined by the position of the lingula and its localization, where an instrument such as the periosteal elevator is tracked from the sigmoid notch inferiorly until resistance is met. Lingulae with greater height are further away from the mandibular foramen, where the inferior alveolar nerve enters, hence providing some form of a safety net during medial horizontal osteotomy. In addition, the variations in the recommended height and depth of needle insertion in inferior alveolar nerve block (IANB) have been attributed to the variations in the height of lingula [46]. This is also critical to avoid direct nerve penetration in IANB, leading to complications such as hematoma and toxicity.

A review of the literature revealed that the distance of lingula to the occlusal plane (LiOP) is in the range of 3.6 mm to 11.22 mm [24,47], with the majority found above the occlusal plane. Interestingly, morphometric variations in the LiOP distance exist within the Turkish population itself. In total, 98.3% of lingulae were located 11.22 mm above the occlusal plane [40]. In this study, 95.4% of lingulae were found 8.55 (3.34) mm above the occlusal plane, 3.6% were at the same level as the occlusal plane, and 1% below the occlusal plane. Similarly, in Koreans, only 1.6% of lingulae were found at or below the occlusal plane [39].

In this study, the LiOP showed significant differences between ethnicities but not between gender and the sides of the mandible. In contrast, a study showed significantly greater LiOP in Chinese males compared to females [47,48]. Sexual dimorphism in Koreans has also been reported in their study investigating internal oblique ridge-guided inferior alveolar nerve block [1]. Based on the findings in this study, it is recommended that the level of needle insertion during IANB be approximately 8 mm above the occlusal plane in Malaysians.

Different anatomical reference points have been used for the measurement between the lingula and mandibular second molar (M2M). These include the distobuccal cusp of the M2M [49], distal side of the alveolar socket [50], cementoenamel junction (CEJ) of the M2M [39], distal side of M2M [42], distobuccal aspect of M2M [17], and the most distal point of the crown of M2M [40]. The lowest value of LiM2M reported was 28.1 ± 2.9 mm [39], while the highest value was 34.57 ± 5.14 mm. In this study, significant differences exist between ethnicities, where Indian adults have the shortest distance of lingulae from the M2M. The reliability of M2M as a stable reference guide or point in providing surgeons with the foresight for localizing and predicting the spatial position of the lingula remains questionable.

LiA, LiP, LiS, LiI

It is undeniable that considerable morphometric variability exists in the mandibular ramus, and its significance may be translated to the clinical setting in procedures such as orthognathic surgeries or inferior alveolar nerve block (IANB). The SSRO has been modified throughout the years to minimize the risk of bad splits and inferior alveolar nerve injury [45,50]. In addition to adding information to the current literature, the results obtained from this study may provide surgeons with a general idea of the position of the lingula for easier and more predictable localization during surgery.

The most recent meta-analysis of ML anatomy reported LiA, LiP, LiS, and LiI to be 16.83 ± 2.66 mm, 15.91 ± 0.64 mm, 15.25 ± 0.84 mm, and 27.86 ± 6.92 mm, respectively [17]. The LiA has been reported to range from 15 to 16 mm and 18 to 20 mm in Caucasian and Asian populations, respectively [2,37,38]. Other than a possible interplay of factors including age, race, and gender, it is worth noticing that different studies employed different reference planes and landmarks, which may contribute to the discrepancies in the measurements obtained. In this study, the measurement of the lingula to the anterior border of the ramus was taken as the shortest distance from the tip of the lingula to the deepest point over the coronoid notch. The coronoid notch along the external oblique ridge was used as the reference landmark for LiA because it is directly visualized intraoperatively during sagittal split ramus osteotomy (SSRO) after raising the mucoperiosteal flap. It was observed that when the IOR acted as the reference point for lingula localization, the antero-posterior ratio was smaller at 45.2% [38], as compared to the norm of 52–55% [28,42]. In this study, the average ratio was higher at 56%, with a significant difference between genders. Malaysian females (56%) recorded a significantly higher ratio than males (54%), indicating a more posteriorly placed lingula in females. This is comparable to the findings in the Korean population [39], where the lingula has been found to be slightly more posteriorly placed in females.

For consistency, it is recommended that the deepest point of the coronoid notch along the external oblique ridge is used as the reference landmark for measurement of LiA, and the depth of the cutting instrument should not exceed 17 to 20 mm when performing medial horizontal osteotomy in the Malaysian population.

As for the distance of the lingula to the sigmoid notch (LiS), the results in this study may provide surgeons with the relevant data to aid in surgical planning and approaches, preventing excessive stripping 14–17 mm beyond the sigmoid notch. One study is of the opinion that dissection of soft tissue on the medial aspect of the mandibular ramus in sagittal split ramus osteotomy could be a factor of neurosensory dysfunction [51]. Hence, by knowing the distance of the ML to different mandibular ramal landmarks, excessive soft tissue stripping at the medial aspect of the ramus can be limited or minimized during sagittal split ramus osteotomy (SSRO), thereby reducing swelling and the risk of nerve injury.

Compared to the data available in the literature, the LiP distance in this study is comparably shorter (14.46 mm), and hence, an intraoral vertical ramus osteotomy should be performed with more caution when the distance is less than 15 mm, as suggested by Sophia et al. (2015) [26].

In IANB, the LiA and LiOP measurements may also be useful in guiding the accurate positioning of needle insertion to achieve successful anesthesia. Based on the findings in this study, the depth of needle insertion should be approximately 17 mm for effective anesthesia. In contrast, a slightly larger needle insertion depth of 20 to 25 mm has been recommended, with consideration given to soft tissue thickness [1].

Generally, it can be observed that the LiA, LiP, and LiS values of the lingula measurement all fall within the reported range in the literature except for the LiI distance, which was shorter in the Malaysian population. Interestingly, morphological and morphometric variations in the ML exist within the same and across different populations, suggesting racial disparities and a possible interplay of factors such as age, race, dentition, skeletal patterns, and environment, other than genetics and ancestry.

Table 9 shows a summary of previous studies performed across different populations. Nonetheless, it is worth noting that different reference planes and anatomical landmarks used may lead to discrepancies in results, limiting direct comparisons between studies.

## 5. Conclusions

The ML is located just slightly superior and posterior relative to the mid-ramus. It is recommended that the cutting instrument is inserted up to approximately 18 mm from the anterior border of the ramus and 8.55 mm above the occlusal plane to reach just posterior and superior to the lingula during medial horizontal osteotomy. In addition to contributing information to the current literature, the results obtained may provide surgeons with a general idea of the position of the lingula for easier and more predictable localization during surgery, especially in cases where the lingula is not easily identified or absent, as in the case of assimilated shape. As the skeletal pattern was not analyzed, the data may not represent or reflect on the actual parameters of subjects who commonly undergo sagittal split ramus osteotomy (SSRO); this is the limitation of the current study.

## Figures and Tables

**Table 1:** Studies of the shape of the lingula summarized.

Study	Population	Type of Study ^1^	Method	No of Samples (Sides)	Gender ^2^	Age ^3^	Shape (*n*, %)					Most Prevalent
							Triangular	Truncated	Nodular	Assimilated	Others	
Tuli et al. (2000) [11]	Indian	DM	Direct visualization	165	131 M; 34 F	NA	226 (68.5%)	52 (15.8%)	36 (10.9%)	16 (4.8%)		Triangular
Kositbowornchai et al. (2007) [16]	Thai	DM	Digital calliper	72 (144)	52 M; 20 F	27–87	24 (16.7%)	68 (47.2%)	33 (22.9%)	19 (13.2%)		Truncated
Jansisyanont et al. (2009) [17]	Thai	DM	Sliding callipers	92 (184)	58 M; 34 F	18–83	55 (29.9%)	85 (46.2%)	36 (19.6%)	8 (4.3%)		Truncated
Lopes et al. (2010) [18]	South Brazilian	DM	Direct visualization	80	NA	NA	66 (41.3%)	58 (36.3%)	17 (10.5%)	19 (11.9%)		Triangular
Samantha and Kharb (2012) [19]	North Indian	DM	Sliding calliper	60 (120)	NA	NA	54 (45.0%)	36 (30.0%)	23 (19.2%)	7 (5.8%)		Triangular
Nirmale et al. (2012) [20]	North Indian	DM	NA	84 (168)	62 M; 22 F	NA	80 (47.6%)	18 (10.7%)	47 (28.0%)	23 (13.7%)		Triangular
Murlimanju et al. (2012) [21]	South Indian	DM	Direct visualization	67 (134)	37 M; 30 F	NA	40 (29.9%)	37 (27.6%)	40 (29.9%)	17 (12.6%)		Triangular and nodular
Varma and Sameer (2013) [13]	South Indian	DM	NA	193 (386)	NA	NA	63 (16.3%)	99 (25.7%)	182 (47.2%)	26 (6.7%)	16 (4.1%) M-shaped	Nodular
Smita (2013) [22]	Indian	DM	NA	50 (100)	NA	NA	42 (42.0%)	36 (36.0%)	10 (10.0%)	12 (12.0%)		Triangular
Sekerci et al. (2013) [23]	Turkish Pediatric	CBCT	NNT software		125 B; 144 G	6–12	74 (13.8%)	126 (23.4%)	260 (48.3%)	78 (14.5%)		Nodular
Sekerci and Sisman (2014) [24]	Turkish	CBCT	NA	412	312 M; 199 F	NA	116 (14.1%)	264 (32.0%)	422 (51.2%)	22 (2.7%)		Nodular
Padmavathi et al. (2014) [25]	South Indian	DM	Vernier calliper	65 (130)	NA	NA	38 (29.2%)	44 (33.8%)	25 (19.2%)	23 (17.7%)		Truncated
Sophia et al. (2015) [26]	South Indian	DM	NA	50 (100)	UNK	UNK	49 (49.0%)	23 (23.0%)	18 (18.0%)	10 (10.0%)		Triangular
Alves and Deana (2015) [27]	Brazilian	DM	NA	132 (253)	165 M; 88 F	NA	59 (23.3%)	124 (49.0%)	67 (26.5%)	3 (1.2%)		Truncated
Senel et al. (2015) [28]	Turkish	CBCT	iCat vision	126	35 M; 28 F	25–70 (46)	28 (22.2%)	24 (19%)	41 (32.5%)	33 (26.2%)		Nodular
Lima at el. (2016) [29]	Brazilian	DM	Calliper	30 (60)	UNK	UNK	11 (18.3%)	29 (48.3%)	-	2 (3.3%)	Rectangular 18 (30.0%)	Trapezoidal
Jung et al. (2018) [30]	Korea	CBCT	PaX-Zenith 3D system (VATECH Co.)	347 (694)	181 M; 166 F	19–50	99 (14.3%)	203 (29.3%)	375 (54.0%)	17 (2.4%)		Nodular
Modasiya and Kanani (2018) [31]	North Indian	DM	Direct visualization	90 (180)	NA	NA	76 (42.2%)	28 (15.5%)	39 (21.7%)	37 (20.6%)		Truncated
Asdullah et al. (2018) [32]	Indian	DM	NA	50 (100)	25 M; 25 F	NA	42 (42.0%)	32 (32.0%)	20 (20.0%)	6 (6.0%)		Triangular
Assis et al. (2019) [12]	South Indian	DM	Direct visualization	50 (100)	NA	NA	47 (47%)	18 (18%)	26 (26%)	7 (7%)	M-shaped 2 (2%)	Triangular
Akcay et al. (2019) [33]	Turkish	CBCT	Mimics software	60 (120)	30 M; 30 F	NA	24 (20.0%)	26 (21.7%)	54 (45.0%)	16 (13.3%)		Nodular
Soares et al. (2019) [34]	Brazilian	DM	Digital calliper	77	NA	NA	35 (45.4%)	33 (42.9%)	-	9 (11.7%)		Triangular
Ozalp et al. (2020) [35]	Turkish (Anatolian)	DM	Digital calliper	50	UNK	UNK	42 (42.0%)	28 (28.0%)	30 (30.0%)	nil		Triangular
Stipo et al. (2022) [14]	Italian	DM	Direct visualization	235 (453)	121 M; 14 F	20-101	49 (10.8%)	175 (38.6%)	119 (26.3%)	18 (4.0%)	Bridge 23 (5.1%) Mixed 69 (15.2%)	Truncated
Elhassan (2025) [36]	Saudi	DM	Digital calliper	100 (200)	80 M; 20 F	NA	118 (59.0%)	44 (22.0%)	26 (13.0%)	12 (6.0%)		Triangular

^1^ DMs: dry mandibles. ^2^ M: male; F: female; NA: not mentioned; UNK: unknown. ^3^ NA: not defined.

**Table 2:** Description of anatomical landmarks, reference planes, and measurements in relation to the mandibular lingula.

Code	Definition
OP	Formed by connecting the midpoint of both mandibular central incisors at the incisal edge and the mesiobuccal cusp of the mandibular first molars (Figure 2)
HLi	Distance of the lingula tip (Li) to the mandibular foramen opening, measured in a vertical line perpendicular to the OP, extending from the tip of the lingula to the lower border (most inferior part of the mandibular foramen opening for assimilated type)
LiOP	Distance of the lingula tip (Li) to the occlusal plane (OP), measured in a vertical line perpendicular to the OP
LiM2M	Distance of the lingula tip (Li) to the mandibular second molar (M2M) cementoenamel junction (CEJ) disto-lingual aspect

**Table 3:** Anatomical landmarks and measurements in relation to the mandibular lingula.

Code	Definition	Description
LiA	Distance of the lingula tip (Li) to the deepest point on the coronoid notch at the anterior border of ramus (A)	Shortest linear distance measured from the anatomical landmarks (irrespective of horizontal or vertical plane)
LiP	Distance of the lingula tip (Li) to the posterior border of ramus (P)	AP − LiA = LiP
LiS	Distance of the lingula tip (Li) to the deepest point of the sigmoid notch at the superior border of the ramus (S)	Shortest linear distance measured from the anatomical landmarks (irrespective of horizontal or vertical plane)
LiI	Distance of the lingula tip (Li) to the inferior border of the ramus	SI − LiS = LiI
AP	Distance from the anterior to posterior border of the ramus; also the antero-posterior diameter of the ramus	Straight line (red line) connecting a point on the deepest concavity of the anterior border of the ramus, passing through the tip of the lingula and extending to the posterior border of the ramus
SI	Distance from the superior to the inferior border of the ramus	Straight line (blue line) connecting a point on the deepest concavity of the sigmoid notch, passing through the tip of the lingula and extending to the inferior border of the ramus
LiA:LiP	Position of the lingula antero-posteriorly in the ramus of the mandible	Ratio of LiA over LiP for localization of the lingula
LiS:LiI	Position of the lingula supero-inferiorly in the ramus of the mandible	Ratio of LiS over LiI for localization of the lingula

**Table 4:** Unilateral and bilateral distribution lingula according to shape.

							Male				Female			
	Total		Bilateral		Unilateral		Bilateral		Unilateral		Bilateral		Unilateral	
	No.	%	No.	%	No.	%	No.	%	No.	%	No.	%	No.	%
Triangular	93	22.6%	54	25.5%	39	19.4%	22	27.2%	12	16.7%	32	24.6%	27	21.1%
Truncated	146	35.4%	65	30.7%	81	40.3%	25	30.9%	38	52.8%	40	30.8%	43	33.6%
Nodular	101	24.5%	61	28.8%	40	19.9%	21	25.9%	8	11.1%	40	30.8%	32	25.0%
Assimilated	71	17.2%	31	14.6%	40	19.9%	13	16.0%	14	19.4%	18	13.8%	26	20.3%
Others	1	0.2%	0	0.0%	1	0.5%	0	0.0%	1	1.4%	0	0.0%	0	0.0%
Total	412		211	51.2%	201	48.8%	81	38.4%	73	36.3%	130	61.6%	128	63.7%

**Table 5:** HLi according to sides, gender, and ethnicity.

Height of Lingula in mm (SD), with 95% CI						
Ethnic		Malay (*n* = 158)	Chinese (*n* = 192)	Indian (*n* = 43)	Overall (*n* = 393)	*p*-Value
Sides	Left (*n* = 169)	5.29 (1.28)	5.35 (1.40)	5.32 (1.46)	5.33 (1.35)	0.727
		5.00–5.59	5.06–5.66	4.60–6.07	5.13–5.54	
	Right (*n* = 172)	5.24 (1.40)	5.22 (1.36)	4.58 (1.12)	5.17 (1.36)	
		4.91–5.59	4.94–5.52	4.05–5.15	4.97–5.41	
Gender	Male (*n* = 127)	5.14 (1.48)	5.37 (1.50)	4.73 (1.39)	5.23 (1.48)	0.061
		4.77–5.57	5.02–5.76	3.79–5.75	4.97–5.49	
	Female (*n* = 214)	5.36 (1.20)	5.25 (1.32)	5.03 (1.33)	5.26 (1.28)	
		5.08–5.63	5.01–5.49	4.45–5.62	5.09–5.43	
	Total	5.26 (1.33)	5.29 (1.38)	4.95 (1.33)	5.25 (1.36)	
		4.97–5.52	5.09–5.44	5.11–5.40	5.11–5.40	

**Table 6:** LiOP according to sides, gender, and ethnicity.

Distance of Lingula to the Occlusal Plane in mm (SD), with 95% CI						
Ethnic		Malay (*n* = 158)	Chinese (*n* = 192)	Indian (*n* = 43)	Overall (*n* = 393)	*p*-Value
Sides	Left (*n* = 201)	9.04 (3.45)	8.85 (3.07)	7.59 (2.37)	8.79 (3.18)	0.063
		8.26–9.82	8.24–9.46	6.51–8.63	8.34–9.23	
	Right (*n* = 198)	8.38 (3.34)	8.55 (3.69)	7.02 (2.87)	8.31 (3.49)	
		7.68–9.17	7.83–9.36	5.78–8.24	7.83–8.83	
Gender	Male (*n* = 76)	9.24 (3.46)	9.53 (3.49)	6.66 (1.77)	9.13 (3.43)	0.479
		8.47–10.02	9.66–10.40	5.66–7.60	8.62–9.68	
	Female (*n* = 124)	8.21 (3.29)	8.32 (3.29)	7.58 (2.90)	8.19 (3.24)	
		7.53–9.03	7.72–8.90	6.54–8.74	7.79–8.61	
	Total	8.71 (3.40)	8.70 (3.39)	7.30 (2.62)	8.55 (3.34)	
		8.17–9.29	8.21–9.21	6.53–8.18	8.24–8.88	

Independent *t*-test.

**Table 7:** LiM2M according to sides, gender, and ethnicity.

Distance of Lingula to the Mandibular Second Molar in mm (SD), with 95% CI						
Ethnic		Malay (*n* = 158)	Chinese (*n* = 192)	Indian (*n* = 43)	Overall (*n* = 393)	*p*-Value
Sides	Left (*n* = 185)	31.78 (3.22)	31.55 (3.78)	29.89 (2.47)	31.45 (3.47)	0.305
		31.02–32.48	30.72–32.39	28.91–30.91	30.93–31.96	
	Right (*n* = 181)	31.92 (3.27)	31.50 (3.81)	29.85 (3.55)	31.49 (3.59)	
		31.21–32.65	30.71–32.37	28.38–31.24	30.97–32.03	
Gender	Male (*n* = 134)	33.12 (3.20)	32.96 (2.96)	30.13 (3.26)	32.75 (3.22)	0.420
		32.37–33.86	32.10–33.71	28.36–31.83	32.17–33.33	
	Female (*n* = 232)	30.77 (2.86)	30.93 (3.94)	29.74 (2.97)	30.73 (3.49)	
		30.19–31.44	30.24–31.59	28.64–30.88	30.29–31.18	
	Total	31.85 (3.24)	31.53 (3.78)	29.87 (3.03)	31.47 (3.53)	
		31.30–32.40	30.96–32.07	28.96–30.90	31.11–31.83	

**Table 8:** LiA, LiP, LiS, and LiI according to gender and ethnicity.

Mean Distance of Lingula to the Anterior, Posterior, Superior, and Inferior Ramus Border in mm (SD), with 95% CI							
Ethnic		Malay (M)	Chinese (C)	Indian (I)	*p*-Value		
					M vs. C	C vs. I	M vs. I
LiA	Male	17.65 (2.44)	18.42 (2.44)	15.83 (1.25)			
		17.16–18.13	17.88–18.96	14.69–16.97			
	Female	17.77 (1.68)	18.28 (2.28)	16.43 (1.96)			
		17.32–18.23	17.91–18.64	15.48–17.19			
	Total	17.71 (2.07)	18.32 (2.32)	16.25 (1.78)	0.022 *	<0.001 *	<0.001 *
		17.40–18.03	18.00–18.64	15.72–16.87			
LiP	Male	15.22 (4.28)	15.50 (3.40)	16.03 (4.17)			
		14.47–15.97	14.66–16.34	14.25–17.80			
	Female	13.98 (2.53)	14.01 (3.37)	13.19 (2.30)			
		13.27–14.68	13.44–14.57	12.02–14.37			
	Total	14.56 (3.51)	14.47 (3.44)	14.05 (3.22)	0.964	0.733	0.64
		14.02–15.10	13.99–14.95	13.10–15.01			
LiS	Male	18.45 (3.01)	17.60 (2.64)	20.66 (4.95)			
		17.80–19.11	16.87–18.34	19.12–22.10			
	Female	17.86 (3.23)	17.15 (2.51)	17.08 (2.96)			
		17.25–18.47	16.66–17.64	16.06–18.10			
	Total	18.14 (3.13)	17.29 (2.55)	18.17 (3.98)	0.017 *	0.161	0.988
		17.66–18.62	16.94–17.65	16.99–19.35			
LiI	Male	29.21 (4.63)	29.42 (4.49)	26.72 (4.66)			
		28.30–30.12	28.40–30.44	24.57–28.87			
	Female	26.53 (3.21)	25.57 (4.15)	25.07 (3.47)			
		25.67–27.39	24.89–26.26	23.65–26.49			
	Total	27.79 (4.15)	26.77 (4.61)	25.57 (3.89)	0.047 *	0.175	0.004 *
		27.15–28.42	26.13–27.41	24.42–26.73			

* one-way ANOVA.

**Table 9:** Summary of studies performed on the lingula measurements to different mandibular anatomical landmarks.

Study	Population	Study Type—Instrument	Ref. Plane	Samples, *n* (Sides)	Gender	Age (Mean)		Lingula Measurements						
								**LiA**	**LiP**	**LiS**	**LiI**	**LiM2M**	**LiOP**	**Hli**
Sophia et al. (2015) [26]	Indian	DM—NA	Mandibular base	50 (100)	UNK	UNK		17.11 ± 2.32	14.86 ± 2.54	18.71 ± 3.18	30.30 ± 5.11	NA	NA	7.45 ± 1.48
Senel et al. (2015) [28]	Turkish	CBCT—iCat	NA	63 (126)	35 M 28 F	25–70 (46)		18.5 ± 2.3	16.9 ± 3.5	18.1 ± 3.6	38.3 ± 5.3	NA	NA	7.8 ± 2.4
Lima at el. (2016) [29]	Brazilian	DM—calliper	NA	30	UNK	UNK	R	18.68 ± 3.75	15.78 ± 2.08	16.64 ± 1.98	33.53 ± 5.23	NA	NA	7.88 ± 2.15
							L	19.96 ± 3.58	15.81 ± 2.55	16.31 ± 2.58	33.87 ± 4.67	NA	NA	7.77 ± 2.01
Alves and Deana (2015) [27]	Brazilian (Amerindian and Caucasian)	DM—digital calliper	NA	132 (253)	165 M 88 F	NA		17.76 ± 2.69	15.28 ± 2.31	17.29 ± 2.57	NA	33.30 ± 4.14	NA	8.29 ± 1.99
Zhou et al. (2017) [39]	Korean	CBCT—OnDemand3 D^®^	Occlusal plane	106 (121)	51 M 55 F	18–36 (26.8)		18.2 ± 2.4	18.2 ± 1.7	15.7 ± 2.7	35.3 ± 3.3	31.0 ± 3.3	6.0 ± 2.9	10.1 ± 2.3
								18.3 ± 2.2	17.0 ± 1.8	15.5 ± 2.3	30.5 ± 2.8	28.1 ± 2.9		9.8 ± 2.1
Tengku Shaeran et al. (2017) [10]	Malaysian	CBCT—NA	NA	51	21 M 30 F	18–35	C I	15.00 ± 2.61	NA	NA	NA	NA	NA	NA
							C III	12.48 ± 2.16	NA	NA	NA	NA	NA	NA
Modasiya and Kanani (2018) [31]	North Indian	DM—vernier calliper	NA	90 (180)	NA	NA		16.62 ± 3.31	15.94 ± 1.63	16.05 ± 2.85	34.16 ± 2.96	NA	7.75 ± 1.81	NA
Akcay et al. (2019) [33]	Turkish	CBCT—mimics	Occlusal plane	60 (120)	16 M 14 F			11.63 ± 1.67	16.18 ± 1.76	18.22 ± 2.81	NA	NA	9.01± 3.18	NA
Zhao et al. (2019) [47]	Chinese	CBCT—NNT	Occlusal plane	407 (814)	201M 206 F	20–35	M	16.53	16.9	16.67	34.74	NA	5.97	NA
							F	16.77	16.42	16.22	32.37	NA	5.03	NA
Jang et al. (2019) [1]	Korean	CBCT—Ez3D-I	Occlusal plane	125	63 M 62 F	15–56	M	14.99 ± 1.27	NA	NA	NA	NA	10.30 ± 2.33	NA
							F	14.37 ± 1.55	NA	NA	NA	NA	7.37 ± 1.94	NA
Ozalp et al. (2020) [35]	Turkish (Anatolian)	DM—digital calliper	NA	50	UNK	UNK		16.86 ± 2.73	14.7 ± 1.6	NA	NA	NA	NA	11.92 ± 2.03
Hsu et al. (2020) [38]	Taiwanese	CBCT—NA	Frankfort horizontal plane	72 (144)	23 M 49 F	NA		19.21 ± 3.02	15.22 ± 2.02	20.04 ± 3.16	31.20 ± 3.81	NA	NA	8.07 ± 2.39
Hayagreev et al. (2021) [48]	Indian	CBCT—Carestream; dicom software	NA	100	NA	<20; >20		16.1 ± 2.2	14.8 ± 2.1	16.3 ± 2.9	32.2 ± 4.0	NA	NA	NA
Lupi et al. (2021) [40]	Italian	CBCT—SimPlant Pro 18^®^	Occlusal plane	111 (201)	56 M 43 F	18–88 (34.9)		16.96 ± 2.40	15.28 ± 2.10	13.87 ± 3.69	31.20 ± 4.35	29.22 ± 3.98	11.22 ± 4.27	NA
Erzurumlu and Torul (2022) [52]	Turkish	CBCT—NA	Occlusal plane	50 (100)	19 M; 31 F	18–56 (31.2)		17.09 ± 2.11	15.46 ± 1.62	17.10 ± 2.57	NA	NA	9.55 ± 2.92	NA
Madiraju and Mohan (2023) [9]	Saudi	CBCT—i-CAT Vision software	NA	125 (250)	68 M; 57 F	16–36 (24.2)		NA	NA	NA	NA	NA	NA	7.73 ± 0.44
Hsu et al. (2024) [44]	Taiwanese	CBCT—RadiAnt	Frankfort horizontal plane	90 (180)	30 M; 60 F	NA		18.88 ± 2.66	15.23 ± 2.02	19.59 ± 3.19	31.34 ± 3.92	NA	NA	NA
Present study	Malaysian	CBCT—Slicer	Occlusal plane	206 (412)	129 M; 77 F	18–60 (33.3)		17.84 ± 2.25	14.46 ± 3.44	17.73 ± 3.00	27.05 ± 4.40	31.47 ± 3.53	8.55 ± 3.34	5.25 ± 1.36

## Data Availability

Data is available upon request.

## Abbreviations

The following abbreviations are used in this manuscript:

CBCT	Cone-Beam Computed Tomograph
ML	Mandibular lingula
MF	Mandibular foramen
IAN	Inferior alveolar nerve
